# ECMO in Cardiac Arrest: A Narrative Review of the Literature

**DOI:** 10.3390/jcm10030534

**Published:** 2021-02-02

**Authors:** Amandine De Charrière, Benjamin Assouline, Marc Scheen, Nathalie Mentha, Carlo Banfi, Karim Bendjelid, Raphaël Giraud

**Affiliations:** 1 Intensive Care Unit, Geneva University Hospitals, 1205 Geneva, Switzerland; amandine.decharriere@hcuge.ch (A.D.C.); benjamin.assouline@hcuge.ch (B.A.); marc.scheen@hcuge.ch (M.S.); karim.bendjelid@hcuge.ch (K.B.); 2 Faculty of Medicine, University of Geneva, 1205 Geneva, Switzerland; nathalie.mentha@hcuge.ch (N.M.); carbanfi@gmail.com (C.B.); 3 Geneva Hemodynamic Research Group, 1206 Geneva, Switzerland; 4 Department of Cardio-Thoracic Surgery Istituto Clinico Sant’Ambrogio, Gruppo Ospedaliero San Donato, Milan, and Chair of Cardiac Surgery, University of Milan, 20149 Milan, Italy

**Keywords:** extracorporeal membrane oxygenation, ECMO, cardiac arrest, ECPR

## Abstract

Cardiac arrest (CA) is a frequent cause of death and a major public health issue. To date, conventional cardiopulmonary resuscitation (CPR) is the only efficient method of resuscitation available that positively impacts prognosis. Extracorporeal membrane oxygenation (ECMO) is a complex and costly technique that requires technical expertise. It is not considered standard of care in all hospitals and should be applied only in high-volume facilities. ECMO combined with CPR is known as ECPR (extracorporeal cardiopulmonary resuscitation) and permits hemodynamic and respiratory stabilization of patients with CA refractory to conventional CPR. This technique allows the parallel treatment of the underlying etiology of CA while maintaining organ perfusion. However, current evidence does not support the routine use of ECPR in all patients with refractory CA. Therefore, an appropriate selection of patients who may benefit from this procedure is key. Reducing the duration of low blood flow by means of performing high-quality CPR and promoting access to ECPR, may improve the survival rate of the patients presenting with refractory CA. Indeed, patients who benefit from ECPR seem to carry better neurological outcomes. The aim of this present narrative review is to present the most recent literature available on ECPR and to clarify its potential therapeutic role, as well as to provide an in-depth explanation of equipment and its set up, the patient selection process, and the patient management post-ECPR.

## 1. Introduction

Cardiac arrest (CA) is a major public health issue. Its incidence in North American and Europe approximates 50 to 100 cases per 100,000 [1]. Cardiovascular etiologies account for half of the cases documented. The 30-day survival rate of out-of-hospital cardiac arrest (OHCA) patients who received cardiopulmonary resuscitation (CPR) is of 10.7% worldwide [2]. Indeed, this poor survival rate has brought interest in the development of a combined approach of conventional resuscitation techniques by means external cardiac compressions and defibrillation with extracorporeal life support by the use of Extracorporeal Membrane Oxygenation (ECMO). Thus, extracorporeal cardiopulmonary resuscitation has become a lifesaving approach for patients suffering a CA that is deemed refractory to conventional resuscitation.

ECPR helps maintain organ perfusion while investigations on the primary etiology of CA are being carried out, and etiologic treatment is being provided. Recently, it has been demonstrated that in-hospital cardiac arrests (IHCA) treated with ECPR show promising survival rates oscillating between 20 to 45% [3,4]. On the other hand, studies performed on non-hospitalized patients (out-of-hospital CA: OHCA) show worse outcomes [5]. Despite this, the most recent guidelines on OHCA’s management elaborate the possibility of using ECPR, but not as routine standard of care. 

The better survival rates post. IHCA are attributed to the earlier implementation of better quality resuscitation, along with quicker access to ECPR. In addition, when looking at studies where ECPR was employed, the length of conventional cardiopulmonary resuscitation (CPR) seems have a negative influence on survival [6]. The differences in survival between OHCA and IHCA handled with ECPR disappear when correcting for the duration of the low-flow period [7]. It would seem therefore plausible that through the promoting of access to ECPR, CPR time will be shortened and survival after CA will be improved [8]. A number of studies have shown the effectiveness of ECPR in cardiac catheterization rooms, emergency departments, and pre-hospital settings [9,10,11]. However, as published in two recent review articles, ECPR programs differ substantially across centers and are a cause of a lack of standardization [12,13]. In this review, the authors present the latest literature on ECPR for patients with CA.

## 2. Methods

This paper provides a narrative review rather than a systematic review of the literature that focuses on the role of ECRP in cardiac arrest refractory to conventional cardio-pulmonary resuscitation. It included articles published in MEDLINE/PubMed database from the year 2000 to the end of October 2020. The search toolbar concentrated on including the following terms: “Extracorporeal membrane Oxygenation” OR “ECMO” OR “ECLS” OR “ECPR” AND “Cardiac Arrest”. A total of 1552 potentially relevant articles were identified. After reading the titles and abstracts, 75 articles were selected for a full analysis. Finally, the references of included papers were screened for additional material not found in the initial literature search and no language restrictions were adopted.

## 3. Place of Implementation of the ECPR

Despite international recommendations, resuscitation procedures vary from one center to the other. The logistics for setting up the ECPR also differ. A number of centers recommend a “scoop and run” approach, with prompt transportation of patients by an ambulance to an ECPR center [9,14]. Alternately, the “stay and treat” attitude, using a mobile emergency and resuscitation unit (SMUR) capable of initiating ECPR on OHCA, has also proven to be an alternative option [10]. Considering the fact that ECPR initiation should be made within 60 min of CA, the best strategy remains to be determined. The facilities and economic health care access of each community plays a predominant role.

The “scoop and run” approach, used in health care facilities with and without an Emergency Medical Service (EMS), has demonstrated limits in the prompt initiation of ECPR [14,15]. In Paris (France), a prehospital ECPR program employed by an EMS has been established in 2011. Some other French cities (Lille Lyon and Perpignan) use a similar program. This approach demonstrated a decrease in low-flow time after OHCA, with similar ECPR initiation times and complications in comparison to hospital-initiated ECPR [10]. However, according to Bougouin et al. [16], among the 13,000 OHCA diagnosed in Paris between 2011 and 2018, 525 benefited from the initiation of ECPR, with 389 being in-hospital and 136 out of hospital. Additionally, there were no differences in mortality between patients who experienced ECPR and those with conventional resuscitation.

The initiation of ECPR requires a specifically trained and well-organized team. In addition, while the ECPR team is concentrated on the cannulation process, a committed team leader must supervise the resuscitation process. The team configuration varies according to local constraints, the organization within the ECPR providing center and the human skill set available. The speed of initiation of ECMO for cardiac indications differs from that required for respiratory failure. Indeed, few cardiac etiologies require rapid treatment initiation for the underlying condition (for example, an acute coronary syndrome (ACS) with percutaneous coronary intervention (PCI)), which, consequentially, considerably reduces the therapeutic time window for circulatory assistance required for survival. For this reason, the choice to implement ECMO has to be made promptly. ECMO as hemodynamic support is ideally carried out in regional referral centers or comprehensive care centers, where it can be implemented as a general management approach for advanced cardiovascular diseases, like ACS requiring PCIs, the implementation of long-term cardiac assist devices and heart transplant [17].

These ECMO centers must have rapidly deployable protocols that quickly bring into play the interveners, being the multidisciplinary heart team, composed of an interventional cardiologists, a cardiac surgeons, heart failure specialists, and the intensivist with all other members of the team deemed essential to the appropriate management strategy [18,19,20]. It is important to highlight that ECMO is a short-term assist device that is used as circulatory support but bears no impact on the etiologic treatment of the underlying condition. The underlying etiology of CA should be managed promptly to maximize the chances of recovery and accelerate safe withdrawal from ECMO. This may include, but is not restricted to, revascularization (percutaneous or surgical) for patients with ACS [21,22], medical or ablative therapy for those with refractory arrhythmia and surgical valve procedures for patients with valvular dysfunction [23,24].

For those who are unlikely to recover sufficient ventricular systolic function or to be safely weaned from VA-ECMO, an early assessment for long-term cardiac support therapy should be considered [25]. Along with advanced cardiovascular platform, ECMO centers that support patients with severe pulmonary vascular disease, should have access to experts in the management of pulmonary arterial hypertension [26]. Finally, patients benefiting from ECMO for cardiac assistance are at risk of developing pulmonary complications requiring the initiation of advanced respiratory support such as a veno-venous ECMO type or even a veno-arteriovenous ECMO. These techniques should be available to the centers that provide these mechanical support techniques [27].

The higher the number of ECMOs implemented in a center, the lower the inhospital mortality [28]. This suggests that high volume ECMO reference centers are likely to have better survival outcomes [29,30,31]. For local and referral centers that do not have the capacity to implement ECMO, we advocate the creation of regional networks around referral and/or comprehensive centers, that are able to deploy ECMO mobile teams to initiate and transport these patients [32]. If ECPR is started by inexperienced local centers in the context of CA, patients may bare substantial risk of suboptimal results. For these centers, we advocate formal collaboration with tertiary care or regional referral centers who are equipped and trained to receive these patients (with common indications, contraindications, cannulation procedure and initiation criteria) [33].

These strategies have been adopted with success for respiratory ECMO centers [30,34,35]. The minimum suitable volume of cases for an ECMO center is still subject of debate. In one study, adult centers dealing with more than 30 ECMO patients per year had a significantly higher survival rate than those treating less than 6 ECMO cases per year (adjusted OR: 0.61, 95% CI 0.46–0.80. The same correlations are observed in ECMOs implemented in the context of heart failure [32]. However, current evidence is based on retrospective data from centers with unspecified levels of expertise.

The use of ECMO in the context of ECPR has its own challenges. In contrast to severe cardiogenic shock, that occurs most often in specific settings (catheterization laboratory, ICU, or operating theater), CA is unpredictable and can occur anywhere inside the hospital, including the emergency department, where ECPR programs are increasingly developing. The ECPR can also be implemented in the prehospital setting. This new approach is currently the subject of an ongoing study (NCT03700125, NCT04620070, NCT02527031) [10,13,36]. It is strongly recommended that ECPR programs be linked to hospital intensive care units which are experienced in managing patients on ECMO and to rapidly transfer patients to a referral center if possible to guarantee appropriate management [37].

## 4. Equipment and Technique for Setting Up ECPR

**Figure 1:**
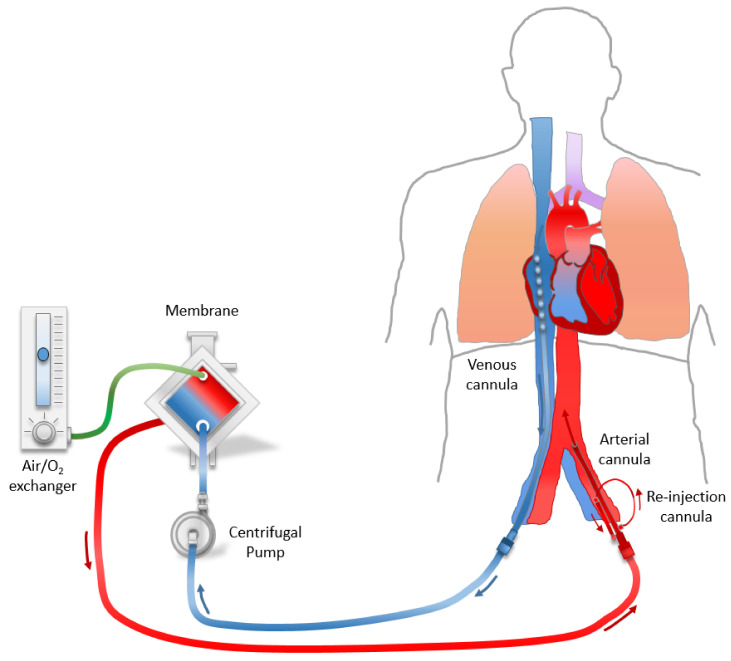
Peripheral fémoro–femoral veno–arterial ECMO used for ECPR.

Placing ECMO during CA is complex and requires specific expertise. The equipment and installations necessary for the practice of ECPR are presented in Table 1. The cannulation of ECMO can be done either percutaneously via ultrasound guided vessel puncture and sequential dilations according to the standard Seldinger technique, or by a direct surgical femoral approach [38]. Surgical approach by incision of the Scarpa triangle is an alternative method. Each technique has its advantages and disadvantages. The details of each technique will not be developed in this article, but the choice of technique essentially depends on the skills of the operator. Figure 1 is a schematic representation of the peripheral fémoro-femoral Veno-Arterial ECMO used for eCPR.

It was nevertheless demonstrated in a retrospective study from a university hospital with a high number of ECMO implantations that among the 814 implanted patients (485 surgical and 329 percutaneous), the percutaneous approach was associated with less local infections (16.5% vs. 27.8%, *p* = 0.001), comparable limb ischemia (8.6% vs. 12.4%, *p* = 0.347), similar neurological complications (2.6% vs. 2.3%, *p* = 0.779), and better 30-days survival rate (63.8% vs. 56.3%, *p* = 0.034). However, percutaneous cannulation (vs. surgical approach) is associated with more post-decannulation vascular complications (14.7% vs. 3.4%, *p* < 0.001), mainly local bleeding requiring surgical hemostasis (9.4% vs. 1.5%, *p* < 0.001) [8]. Whichever the cannulation technique used, echocardiography is mandatory to certify that the guides and cannulas are correctly placed before the ECMO is installed [39].

The size of the cannulas is a crucial determinant of the effectiveness of ECPR. The appropriate choice of the diameter of the venous cannula allows for optimal drainage of the patient’s blood. The correct diameter of the arterial cannula guarantees a satisfactory reinjection of blood to the patient [38]. In adults, a minimum of 23 to 25 Fr for the drainage cannula and 17 to 19 Fr for the reinjection cannula are recommended. This is despite the lack of evidence regarding the ideal ECMO flow needed for maintaining good organ perfusion [38].

The arterial cannula can completely block the femoral artery and cause ischemia of the cannulated lower limb. To prevent the occurrence of such a complication, it is advocated to systematically place a reperfusion cannula in the ipsilateral superficial femoral artery. This reperfusion cannula is connected to the arterial circuit and thus allows adequate perfusion of the distal end of the cannulated lower limb. The placement of this reperfusion strategy can be done at a distance from the initial cannulation, with early placement being favored. This reperfusion catheter can be inserted either surgically or percutaneously under ultrasound guidance [40]. An hourly Doppler monitoring of the foot perfusion should then be performed by an ICU nurse.

## 5. Patient Selection Process

Until recently, refractory CA used to be defined as a CA that does not respond to 30 min of conventional cardiopulmonary resuscitation (CPR) [41]. The choice to move from conventional CPR to ECPR is frequently made late, after an average of 30 to 40 min of unsuccessful CPR. For this reason, survival rates are highly variable.

Convincing evidence points to the length of conventional CPR as being an independent prognostic parameter for refractory OHCA. The longer the conventional CPR, the worse the outcome, and this period of CA is called the period of low flow [7]. Optimally, ECPR should be started within 60 min of the start of the CA so that the period of low flow is maintained below 60 min [4]. Kim et al. suggested that the optimal time to switch from conventional CPR to the initiation of ECPR is 21 min [42]. Reynolds et al. have shown that the probability of survival with favorable neurological outcomes decreases after 16 min of CPR [43]. Therefore, for eligible patients who have not responded to the first 10 min of conventional resuscitation, ECPR should be anticipated and made instantly available. Moreover, the ECPR should be initiated within 20 min of the CA so that the patient can be assisted by the ECMO as soon as possible. However, the most important determinant in terms of survival is the duration of no-flow, during which time the patient receives no resuscitation [44]. Current recommendations point out that early high-quality cardiac compressions influences the effectiveness of all other procedures [45]. It is therefore essential that CPR should be started immediately after the collapse in order to minimize the no-flow period.

Even if the upper age limit for ECPR differs, most studies rule out patients over the age of 70 to 75 [5,46,47,48]. Shockable heart rhythms are associated with lower mortality in OHCA patients [49]. The initial heart rate can also be predictive of a shorter duration of no-flow. In a recent study, Tanguay-Rioux et al. showed for 2532 OHCA that global survival was 13.8% to 34% for shockable initial rhythms. The probability of maintaining a shockable initial rhythm diminished with increasing no-flow duration (adjusted OR: 0.88 per minute, 95% CI 0.85–0.91). In patients with initial shockable rhythms, 94% (95% CI 92–96%) had a no-flow of less than 10 min. The authors conclude that the chances of having a shockable initial rhythm decreases with each additional minute of no-flow, emphasizing the importance of having early access to defibrillation and the necessity to screen early for potential candidates for ECPR [50].

On the other hand, patients with a low-flow > 90 min are less likely to benefit from ECPR [4]. Indeed, the latest recommendations suggest that ECPR should be started within the first 60 min of the CA [45]. In a recent retrospective study on 135 patients in refractory CA with the implementation of ECPR, Otani et al. studied the prognostic factors that predicted a favorable neurological outcome. Among the patients included, 22 (16%) had a satisfactory neurological prognosis. The low-flow was shorter in the “satisfactory neurological evolution” group with a threshold of 58 min [51]. 

It is essential for CPR be of good quality during the low-flow period [52]. To ensure this, monitoring expired CO_2_ (EtCO_2_), a validated indicator of survival in CA, is recommended. EtCO_2_ < 10 mmHg appears to be associated with a lower survival rate.

Ventilation during CPR may lead to an increase in peak inspiratory pressures, with high inspiratory pressures potentially becoming a source of lung injury. The latter makes it challenging to deliver the required tidal volume for adequate ventilation. The utilization of mechanical compression devices further contributes to the difficulties faced by the medical providers. However, none of the current international guidelines provide recommendations on the “best” mechanical ventilation strategy to use during mechanical CPR. A recent review of the literature on 38 papers exploring the various ventilation strategies during mechanical CPR, demonstrated that a high FiO_2_ must be guaranteed during CPR and, with a lower grade of evidence, that turning off inspiratory triggering and applying PEEP ≥ 5 cm H_2_O might be beneficial. In the review, the author also presented an interesting operating algorithm that may be worthy of future discussion and perhaps a prospective trial [53].

In summary, the proper selection of patients who can benefit from ECPR in the event of a CA is essential. It seems rational to select patients having no known major comorbidities, a persistent shockable heart rate, the shortest possible no-flow time and to rapidly implement high-quality CPR with a target EtCO_2_ > 10 mmHg during resuscitation. More recently, “signs of life” (spontaneous movements, breathing, gasping and pupillary reflex), independent of the cardiac rhythm, have also been proposed as good predictors of survival in patients having benefited from ECPR [9]. Finally, the specific cases of refractory CA in the context of accidental hypothermia must be the subject of specific management with a specific protocol where the ECPR can play its role [54,55].

ECPR should only be used in highly selected patients with a cardiac origin of arrest. Moreover, indications and contraindications may vary according to hospital, experience level of the cardiac arrest team, and readiness of ECLS deployment. To date, there has been a lack of RCTs of ECPR and there are no prospectively validated criteria for ECPR indications or patient selection. However, favorable outcome can be expected with ECPR when employed for cardiac arrest under several conditions (Table 2) [45,56].

## 6. Patients Management after ECPR

Management after ECPR is focused on preserving adequate organ perfusion restoring a pulsating rhythm with a native cardiac output. After establishing an adequate extracorporeal circulation, the chest compressions may be stopped. At this point, after an improvement in coronary perfusion pressure and a better supply of oxygen from the extracorporeal pump, defibrillation of shockable rhythms is generally more effective. Managing hyperoxia is challenging after the introduction of extracorporeal circulation. The oxygen supply has to be adequately calibrated in order to not negatively impact neurological and cardiovascular outcomes. The mean arterial blood pressure (MAP) should be maintained between 65 and 75 mmHg (expert recommendation) with a careful balance between flow and negative pressure inside the venous cannula. Most of the time, vasopressors (noradrenaline) are used to reach the target MAP. Invasive blood pressure monitoring is mandatory. It is advisable to catheterize the right radial artery in order to anticipate the occurrence of Harlequin syndrome in case of recovery of the left ventricular function and to allow the detection of hypoxemia of pulmonary origin. Sometimes aggressive volume resuscitation (ischemia-reperfusion syndrome) may be necessary to ensure an adequate preload to support ECPR.

The circulatory support by peripheral veno-arterial ECMO (VA-ECMO) is based on organ perfusion via retrograde arterial flow [57]. An important limitation of this strategy is the increase in the left ventricular afterload [58]. In the context of cardiogenic shock, a condition that often presents after refractory CA, an increase in the left ventricular afterload can lead to an increase in myocardial ischemia, an increased incidence of ventricular arrhythmias, pulmonary edema, and thrombotic events [59,60,61,62]. Severe aortic regurgitation should be a contraindication to VA-ECMO because the risk of left ventricular overload is too high. Moreover, for mild to moderate aortic regurgitation, the risk of ventricular distension is not negligible [63]. Several interventions can be used in conjunction with ECMO to unload the left ventricle (LV) and thereby avoid some of these complications related to an increase in LV afterload [57,61]. However, the optimal approach to decrease left ventricular afterload during VA-ECMO remains unknown. Inotropic drugs, like dobutamine, can be given in small doses to ensure the opening of the aortic valve and minimal output of the left ventricle [64]. The later optimizes left ventricular contractility with the opening of the aortic valve and prevents the occurrence of acute congestive pulmonary edema A minimal pulsed pressure of at least 10 mmHg is recommended. In some centers, the placement of an intra-aortic balloon pump is considered standard of care, while in others the assessment of LV unloading dictates its use [65].

Finally, certain research groups have shown that the unloading of the LV via a continuous axial flow pump such as the Impella^®^ type improves the survival of patients with VA-ECMO [66]. In a recent meta-analysis of almost 4000 patients, 42% of whom received a concomitant left ventricular unloading device with VA-ECMO (intra-aortic balloon 91.7%, percutaneous ventricular assist device 5.5%, pulmonary venous cannulation or left atrial trans-septal 2.8%), the mortality was lower in the patients having benefited from a ventricular unloaded device compared to the patients not having benefited from it. (54% vs. 65%, relative risk: 0.79; 95% confidence interval: 0.72 to 0.87; *p* < 0.001). However, rates of hemolysis were higher in patients with a left ventricular unloading device [67].

Once the patient is assisted and stabilized on VA-ECMO, treatment of the suspected cause of the CA should be initiated. If an acute coronary syndrome is suspected, the patient has to be referred to for immediate coronary angiography with PCI. In this specific group of patients, studies have demonstrated that coronary lesions are frequently multiple and proximal [68,69]. Moreover, it has been shown that the delay between CA and PCI is associated with survival [70]. If a pulmonary embolism is the origin of the CA, an injected pulmonary CT-scan should be considered to confirm the diagnosis [71]. Echocardiography can also provide useful diagnostic clues [72]. Some teams also recommend ECMO support to perform in situ thrombolysis or surgical thrombectomy [73,74]. Others believe that ECMO’s effect is solely attributable to the patient’s intrinsic fibrinolysis and that therefore, patients should be managed with heparin therapy only [75,76,77]. Lastly, intracranial hemorrhage (ICH) is a common complication in adults treated with ECMO and associated with increased mortality. Treating an ICH during ECMO represents a balance between pro- and anticoagulatory demands. Neurosurgical treatment is associated with severe morbidity, but has been successful in selected cases [78]. If an ICH is suspected, a cerebral CT-scan must be the first priority over any subsequent interventions or ECMO insertion.

## 7. Neurological Outcomes

Regardless of the heart rate at the time of cannulation, ECPR optimizes the organ perfusion of patients suffering from refractory CA due to ventricular fibrillation and/or tachycardia (VF/VT). By achieving hemodynamic stability, ECPR makes it possible to halt the evolution of ischemic lesions without necessarily obtaining a return of spontaneous circulation (ROSC). It therefore provides time to correct the severe metabolic disturbances that develops during prolonged CPR and makes it possible to treat the underlying etiologies that can perpetuate refractory VF/TV. These stabilization strategies are associated with improved survival and satisfactory neurological outcomes in patients with refractory CA [10,69,79]. Furthermore, ECPR is able to stabilize the patient at a constant temperature of 36 °C for 24 h [80].

In the ECPR cohort study of the University of Minnesota, 100% of the patients who benefited from the association of CPR (lasting between 20 and 29 min) before the initiation of ECMO survived with a satisfactory neurological outcome. The results were grimmer in the conventional CPR group where only 24% of the patients survived with a satisfactory neurological outcome. In comparison to the conventional CPR group, ECPR demonstrated survival benefits from CPR durations of up to 98 min. Ischemic injury before the onset of ECMO seems to be the determining factor in predicting prognosis. In this same cohort, a 25% drop in survival rates was found for every 10 min time lapse beyond 29 min of CPR [81]. Previous studies have also demonstrated a link between the duration of CPR and survival during ECPR [4,16,82].

ECPR can improve survival after prolonged CPR, but the avoidance of harm to those who would have otherwise survived with conventional CPR alone should be a concern. Recent studies on patients with OHCA benefiting from conventional CPR have shown that 99% of surviving patients with a satisfactory neurological state underwent ROSC within a maximum of 28 to 39 min of CPR performed by healthcare professionals [83,84,85,86].

Most ECPR programs require the transportation of patients to a hospital for implantation of ECMO. It is therefore vital to estimate the timing of an indication for the transfer. Indeed, transferring a patient in cardiac arrest could reduce the effectiveness of resuscitation, potentially preventing the survival of some patients. Reynolds et al. [85] studied the relationship between advanced therapies and the risk of transport in patients meeting ECPR criteria collected from observational studies. They included patients between 18 and 65 years of age, with cardiac arrests occurring in the presence of witnesses, CPR initiated within 10 min and the absence of asystole as an initial heart rhythm. They found that 90% of survivors with a favorable neurological outcome had a ROSC within 21 min, and that the probability of survival with satisfactory neurological outcome if CPR was prolonged beyond 20 min is of 8.4%. The authors recommend that 21 min of standard resuscitation as a cut-off before transport for ECPR.

In clinical practice, it seems reasonable to recommend the immediate transport of patients with cardiac arrest who do not respond to the first resuscitation measures. Indeed, the carrying out of the first specialized resuscitation measures corresponds approximately to 10 min in the European recommendations. It is therefore suggested that this period of time should be used to consider the transport for ECPR. Adding the shortest timing between «transfer decision» and «effective transfer» brings the timing to transport to approximately 20 min of CPR as is previously mentioned.

Some centers recommend performing a chest compressions using an automatic compression board. However, in a recent meta-analysis, the level of evidence does not suggest that CPR algorithms including mechanical chest compression devices are superior to the conventional manual chest compression technique. Mechanical chest compressors used by trained medical providers are a reasonable alternative to manual chest compressions in situations where high-quality manual chest compressions are not feasible or hazardous (for example, few lifeguards available, prolonged CPR, during a hypothermic CA, in an ambulance, in the angiography room, or during preparation for an ECPR) [87]. Moreover, other investigators showed that chest compressions while moving in-hospital CA patients performed on a stretcher equipped with the wing method can produce high-quality chest compressions [88].

The time race for successful ECPR has important implications for the implementation of such protocols. With current prehospital resuscitation techniques, recommendations suggest an optimal time lapse of approximately 30 min of CPR before the implementation of ECMO for refractory OHCA. However, the survival benefits of ECPR may extend beyond 60 min. Therefore, ECPR programs should aim to maximize the number of patients who can be cannulated in less than 30 min without necessarily excluding patients with longer resuscitation times.

The future optimization of prehospital care could also improve the survival associated with ECPR. Prehospital CPR strategies that improve the perfusion achieved by CPR or that reduce the patient’s metabolic demand can prolong the time of effective CPR, thus delaying the onset of ischemic damage. The prehospital initiation of ECPR could also provide a rapid stabilization. To date, the largest study on the application of ECPR in patients presenting with OHCA has recently been published. It gives new information on the effectiveness of this strategy. Bougouin et al. [16] reported more than 13,000 cases of OHCA in the Paris metropolitan area. Of the 12,396 patients treated with conventional CPR, 8.6% (1061) survived to discharge from the hospital compared with 8.4% (44) of the 523 ECPR patients. ECPR was tried but failed in 11% (58) of patients. Factors favoring survival in the ECPR group include a transient return to spontaneous circulation (ROSC) and an initial shockable rhythm before ECPR. It should be noted that prehospital ECPR is correlated with higher survival (OR 2.9, 95% CI 1.5–5.9, *p* = 0.002) and more favorable neurological outcomes (OR 2.9, 95% CI 1.3–6.4, *p* = 0.008) compared to patients receiving ECPR after admission at the hospital. 

However, this study has many limitations, including a selection bias. The decision to initiate ECPR was taken at the discretion of each clinician and not according to a strict pre-established algorithm, and thus provides a plethora of potential confounding factors. This is shown by the differences in baseline descriptions of the ECPR patients. Patients were younger and more predisposed to benefit from CPR by witnesses (81% vs. 49%, *p* < 0.001) but, more relevantly, these received a prolonged CPR longer than 30 min (99% vs. 77%, *p* < 0.001). The authors attempted to correct for the known confounding factors via a multivariate analysis (OR 1.3, CI 95% 0.8–2.1, *p* = 0.24) nor in the propensity analysis (OR 0.8, 95% CI 0.5–1.3, *p* = 0.41) but were unable to determine whether ECPR was correlated with improved survival in the hospital setting. There are numerous differences in the study subgroups, particularly among patients without ROSC and those with non-shockable rhythms. ECPR may potentially show different outcomes among these subgroups, perhaps to be investigated in the future with a dedicated study [16]. 

More relevantly, neurological outcomes and long-term quality of life were not examined. It would have been desirable not to limit the analysis to hospital mortality and to analyze factors such as functional recovery and long-term survival with acceptable neurological sequelae [89,90]. This study will remain a pilgrim to the believers of mechanical support devices and the role they may play in improving outcomes during cardiac arrest. It will stimulate further research in the area in order to remedy the poor results observed in patients experiencing OHCA. The fact that there are no statistically significant differences in survival between patients who benefited from ECPR and those managed with conventional RCP requires a reassessment of the role of ECPR in patients with OHCA. This last publication did have a number of qualities, including the high number of patients included, the functional experience of the teams involved to facilitate the prompt implementation of ECPR, and its multicenter observational design, offering “real” data. Finally, ECPR is a form of mechanical support that requires a particularly complex and large organization of human and technical resources. It also requires a very high level of expertise from the practitioners performing the cannulation under extreme conditions. It is therefore essential for the maintaining of these types of programs to insure a sufficient number of interventions and allowing high exposure of the professionals involved, to maintain a high quality of standard of care.

## 8. Conclusions

CA remains a frequent cause of death and a major public health issue. Conventional CPR is to date the sole efficient resuscitation procedure available to improve the prognosis of these patients. ECMO is a complex and relatively high-priced technique that necessitates expertise. Therefore, it cannot be used in all hospitals and has to be performed in high-volume centers that routinely perform these procedures. ECPR allows for hemodynamic and respiratory stabilization of patients with CA refractory to conventional CPR and permits, by means of preserving organ perfusion, the initiation of treatment of the underlying cause of CA. However, the current evidence does not support a recommendation for routine use of ECPR in all patients with refractory CA. Therefore, it seems crucial to appropriately select the patients among those who could potentially benefit from its use. This may include patients presenting with a risk of imminent death with specifically designed scores that can predict a survival benefit associated with the use of ECPR. The desirable benefit of its use will be adequate resuscitation which promotes medium to long term survival acceptable neurological outcomes. Finally protocols to best manage patients with refractory CA by means of extra hospital ECPR remain an active area of research.

## Figures and Tables

**Table 1:** Equipment and installation to implement ECPR (extracorporeal cardiopulmonary resuscitation).

Percutaneous ECMO insertion kit
Vascular ultrasound probe with sterile protection
Echocardiography with Doppler mode
ECMO pump compatible with transportation
Backup ECMO pump
A battery that can last 45 min minimum
Clamps for cannulas/circuit
Surgery instruments for any hemorrhagic complications
Additional light for surgery
Heater for ECMO
Equipment for in-hospital transportation
ECMO pump transportation Cart
Emergency bag with all drugs and clamps
ECMO already primed available 24 h/24
Vascular Doppler or NIRS to check distal perfusion of the leg
Fiberoptic bronchoscope
Any device able to unload the left ventricle (IABP, Impella®)

ECPR: extracorporeal cardiopulmonary resuscitation, ECMO: Extracorporeal membrane oxygenation, NIRS: Near Infrared Spectroscopy, IABP: Intra-aortic balloon pump.

**Table 2:** Favorable and unfavorable criteria for initiating an in-hospital ECPR for an out-of-hospital cardiac arrest.

Favorable Criteria
Refractory CA with reversible cause
Witnessed CA
High quality CPR started immediately
Age < 70
Initial shockable rhythm
EtCO2 > 10 mmHg
Signs of life under CPR
Reaching hospital < 40 min allowing ECMO initiation < 60 min. (Maximum low-flow < 60 min)
Pulse perceived under CPR
**Unfavorable Criteria**
CA without any witnesses and/or ignorance of the duration of the no-flow
No-flow > 3 min and/or Low-flow > 90 min
Poor quality CPR (EtCO2 < 10 mmHg and/or absence of pulse perceived under massage)
Non-shockable initial rhythm
Major comorbidities
Obvious clinical signs of irreversible death (rigidity, lividity)

ECPR: Extracorporeal cardiopulmonary resuscitation, CA: Cardiac arrest, CPR: Cardiopulmonary resuscitation, EtCO_2_: End-tidal CO_2_, ECMO: Extracorporeal membrane oxygenation.

## Data Availability

Non-applicable.
